# Depressive Symptoms, Systemic Inflammation, and Survival Among Patients With Head and Neck Cancer

**DOI:** 10.1001/jamaoto.2024.0231

**Published:** 2024-03-28

**Authors:** Elizabeth Cash, Christy Albert, Iona Palmer, Baylee Polzin, Alyssa Kabithe, Devaughn Crawford, Jeffrey M. Bumpous, Sandra E. Sephton

**Affiliations:** 1 Department of Otolaryngology–Head and Neck Surgery and Communicative Disorders, University of Louisville School of Medicine, Louisville, Kentucky; 2 University of Louisville Healthcare−Brown Cancer Center, Louisville, Kentucky; 3 University of Louisville School of Medicine, Louisville, Kentucky; 4 Department of Psychology, Brigham Young University, Provo, Utah; 5 Department of Psychological and Brain Sciences, University of Louisville, Louisville, Kentucky

## Abstract

**Importance:**

Patients with head and neck cancer experience high rates of depression. Depression and systemic inflammation have been found to be associated in numerous cancer types, often independently from disease status. Depression-related inflammation may elevate the risks for poor tumor response to treatment and early mortality, and comprises a mechanism by which depression is associated with survival in head and neck cancer.

**Objective:**

To assess mediation pathways incorporating pretreatment depressive symptoms, pretreatment inflammation, and tumor response posttreatment on overall survival among patients with head and neck cancer.

**Design, Setting, and Participants:**

This was a prospective observational cohort study of patients with head and neck cancer treated in a single multidisciplinary head and neck cancer clinic from May 10, 2013, to December 30, 2019, and followed up for 2 years. Data analysis was performed from June 29, 2022, to June 23, 2023.

**Exposures:**

Patient-reported depressive symptoms using the Patient Health Questionnaire−9 item (PHQ−9) at treatment planning; pretreatment hematology workup for systemic inflammation index (SII) score; and clinical data review for tumor response (complete vs incomplete) and overall survival.

**Main Outcomes:**

Two-year overall survival.

**Results:**

The total study cohort included 394 patients (mean [SD] age, 62.5 [11.5] years; 277 [70.3%] males) with head and neck cancer. Among 285 patients (72.3%) who scored below the clinical cutoff for depression on the PHQ−9, depressive symptoms were significantly associated with inflammation (partial *r*, 0.168; 95% CI, 0.007-0.038). In addition, both depression and inflammation were associated with early mortality (PHQ−9: hazard ratio [HR], 1.04; 95% CI, 1.02-1.07; SII: HR, 1.36; 95% CI, 1.08-1.71). The depression-survival association was fully mediated by inflammation (HR, 1.28; 95% CI, 1.00-1.64). Depressive symptoms were also associated with poorer tumor response (odds ratio, 1.05; 95% CI, 1.01-1.08), and the depression-survival association was partially mediated by tumor response (HR, 9.44; 95% CI, 6.23-14.32). Systemic inflammation was not associated with tumor response.

**Conclusions:**

In this cohort study, systemic inflammation emerged as a novel candidate mechanism of the association of depression with mortality. Tumor response partially mediated effects of depression on mortality, replicating prior work. Thus, depression stands out as a highly feasible target for renewed clinical attention. Even mild symptoms of depression during the treatment-planning phase may be associated with higher systemic inflammation in addition to poorer tumor response to treatment and survival outcomes; therefore, depression should be clinically addressed.

## Key Points

**Question:**

Does systemic inflammation mediate the association between depression at diagnosis and subsequent 2-year overall survival among patients with head and neck cancer?

**Findings:**

This cohort study of 394 patients assessed depressive symptoms and systemic inflammation in the diagnostic period, tumor response 6 months posttreatment, and 2-year overall survival, and found that depression-survival association was fully mediated by systemic inflammation and partially mediated by tumor response.

**Meaning:**

These findings indicate that even mild symptoms of depression during the treatment-planning phase may be associated with higher inflammation, poorer treatment outcomes, and higher mortality in patients with head and neck cancer, and should be clinically addressed.

## Teaser

This cohort study assessed the associations of pretreatment depressive symptoms and inflammation with posttreatment tumor response and overall survival among patients with head and neck cancer.

## Introduction

Depression may affect as many as 1 in 3 patients with head and neck cancer.^1,2^ Depression is a known prognostic indicator of early head and neck cancer mortality and in the diagnostic phase appears to erode clinical treatment success.^3^ Specifically, this research group found depression to be prognostic for 2-year overall survival, independent of age, tumor stage, and smoking history.^3^ The depression-survival relationship was observed even among patients with mild depressive symptoms and those who evidenced a complete response to curative cancer treatment.^3^

**Figure 1.**
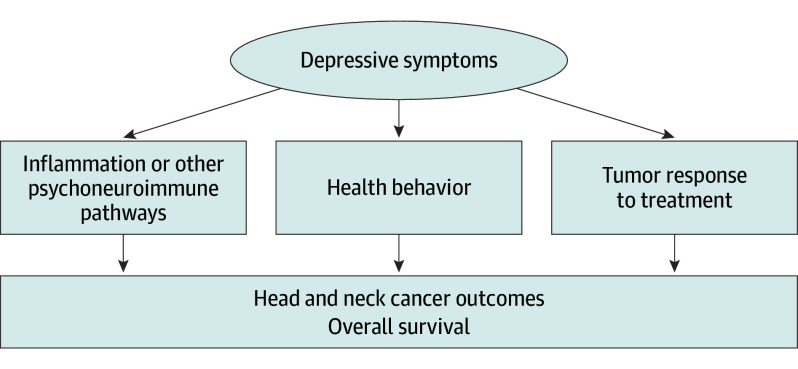
Conceptual Model of the Influence of Depression on Head and Neck Cancer Outcomes There are 3 pathways by which depressive symptoms may affect subsequent cancer survival outcomes: (1) via psychoneuroimmune effects including inflammation that can interfere with tumor defense mechanisms; (2) through behavioral pathways, eg, effects of depression on patient health behaviors and treatment adherence; and (3) through biological effects on tumor response to treatment.

Inflammation is 1 of 3 plausible mediators connecting depression and cancer progression (Figure 1). Conclusive data demonstrate an association of systemic inflammation with depression across a wide array of patient populations.^4,5^ Among patients with cancer, inflammation has frequently been associated with both tumor progression and depression. Bidirectional brain-immune relationships are marked by elevated blood levels of inflammatory cytokines (eg, IL-6, TNF-α).^6,7,8^ The pathophysiologic mechanisms connecting these factors is under investigation.^9^ Elevated inflammatory cytokines have been noted as a prelude to depressive episodes among people with major depressive disorder^10,11^ and are believed to contribute to mechanisms of depression onset and poorer antidepressant treatment response.^12^ Depression may also be associated with inflammation through health behavior relevant to cancer progression (Figure 1). Tobacco use after cancer diagnosis, poor nutrition, poor sleep, and insufficient stress-management, self-care, and exercise can perpetuate inflammatory processes and affect tumor resistance capabilities.^13^ Chronic systemic inflammation can promote tumor progression by interfering with the action of some antitumor drugs, stimulating angiogenesis and tumor cell proliferation, and by downregulating some aspects of antitumor immunity.^14^ Highlighting the clinical relevance of these data, the systemic inflammation index (SII) has been posited as a biological indicator of depressive disorders.^15,16^ This biomarker is used as a prognostic factor in diseases with inflammation-related causes.^17^ The SII has also recently been highlighted as a predictor of mortality for head and neck as well as other cancers.^18,19,20,21,22,23^

This study aimed to replicate our prior work implicating depressive symptoms as a significant prognostic factor for head and neck cancer survival^3,24^ among a separate and larger patient population. We sought to extend prior work by evaluating associations of systemic inflammation with tumor response to treatment and overall survival. We hypothesized that patients with head and neck cancer who presented at treatment planning with greater depressive symptoms would experience significantly shorter overall cancer survival, and assessed whether this association might be mediated through 2 biological pathways: systemic inflammation and tumor response to treatment. We also hypothesized that greater systemic inflammation would be significantly associated with poorer tumor response to treatment.

## Methods

This study was reviewed and approved by the institutional review board of the University of Louisville, and informed consent was waived because data collection for research purposes was conducted retrospectively. The study followed the Strengthening the Reporting of Observational Studies in Epidemiology (STROBE) reporting guideline.

### Participants and Procedures

For this prospective cohort, we reviewed all patients presenting to our institutional multidisciplinary head and neck cancer clinic from May 10, 2013, to December 30, 2019. Patients completed depression assessments at presentation to the multidisciplinary clinic for surgery, radiation therapy, and/or chemotherapy treatment planning. Typically, patients had received a notification of a biopsy specimen−proven diagnosis 1 to 4 weeks before presentation, with 17% of the study participants already having undergone a surgical procedure to confirm the extent of malignant neoplasm or for extirpation.

In all, 804 patients who were referred to our clinic completed the depression assessment during the 6.5-year study period. We excluded 410 patients because they either presented with nasopharyngeal disease; nonsquamous cell pathology; recurrent or metastatic disease or a synchronous primary cancer in another site; were treated with palliative intent; or were missing follow-up data because they continued treatment at another location for reasons such as convenience.

### Measures

Data were collected in 3 waves. Wave 1 of data collection began at the time of patient presentation to a multidisciplinary head and neck cancer clinic for treatment planning during which depressive symptoms were assessed. Systemic inflammation was assessed either at that same visit or within a 4-week time frame. Wave 2 occurred 6 months after completion of cancer treatment (approximately 8 months after wave 1) for assessment of tumor response. Wave 3 occurred 2 years later, at which point overall survival was assessed.

#### Depression Assessment

The Patient Health Questionnaire−9 item (PHQ−9)^25^ was administered to indicate frequency of depressive symptoms during the previous 2 weeks on a scale from 0 to 3. The PHQ−9 has demonstrated adequate internal consistency, reliability, and validity,^25^ and has been used for self-reported assessment among patients with head and neck cancer.^26,27^ Sum scores were generated for all 9 items (range 0-27).

#### Clinical Variables

Patient demographic characteristics including age, sex, and alcohol and smoking history were collected from clinical intake forms. Cancer stage classification (T classification) was determined using the *American Joint Commission on Cancer Staging System, eighth edition*, and all available clinical, pathologic, and radiographic data. The viral status of oropharyngeal cancer cases was determined from either human papillomavirus tested by in situ hybridization or p16 (immunohistochemistry) results. Tumor location was classified into 5 categories: oral, oropharyngeal, laryngeal, hypopharyngeal, and other (eg, neck disease/tumors of unknown origin); all were confirmed pathologically to be squamous cell type.

A routine complete blood count (CBC) was obtained from a blood sample drawn either preoperatively or postoperatively and in all cases before initiation of systemic treatment; from this sample, the SII was calculated (absolute neutrophil count** × **platelet count / absolute lymphocyte count).^23^ Some patient records had missing or incomplete CBC data; SII was calculated for the 292 patients (74%) with complete data. For another 36 patients, CBC data were not available until after treatment initiation; however, statistical comparison of SII values for patients whose workup was completed before treatment initiation vs during treatment did not reveal any significant differences; therefore, these 36 patients were included in tests of hypotheses.

Medical records were reviewed 6 months after all participants had completed treatment, yielding data on treatment modality and the clinical response of the tumor to treatment. A dichotomous indicator of tumor response was calculated: patients were coded as having an incomplete tumor response when there was clinical or radiological evidence of persistent disease; if recurrence, metastases, or a new primary cancer was observed; or when early death precluded evaluation at 6 months posttreatment, according to RECIST guidelines, version 1.1.^28^ Otherwise, patients were coded as having a complete response to treatment. Two-year overall survival data was then collected and calculated from the date of study entry, which was also the date of the patient’s presentation to the multidisciplinary clinic.

### Statistical Analyses

Descriptive and summary statistics characterized clinical and demographic features. Independent variables were centered^29^ and all assumptions were met.

#### Primary Analyses

Associations of depressive symptoms, SII, and tumor response with overall survival were each determined using separate Cox proportional hazards models. A multiple regression analysis tested the association between depressive symptoms and SII. Separate logistic regressions tested the association between depressive symptoms and tumor response, and the association between SII and treatment response.

Tests of mediation to evaluate the roles of both SII and tumor response in the association between depression and survival were performed using the MacArthur approach.^30^ This approach allows for the assessment of mediation effects in the context of survival. A Cox model was constructed to include entry depressive symptoms, SII, and their interaction. A second Cox model was constructed to include entry depressive symptoms, tumor response assessed at the 6-month follow-up, and their interaction term.

#### Secondary Analyses

To identify possible confounders, Spearman rank correlations assessed the contribution of traditional prognostic indicators, including cancer stage, treatment modality, site of disease, viral status, age at diagnosis, and sex. Those that correlated with both the independent and dependent variables (eg, both depression and survival) were considered possible confounds to hypothesized relationships.^31^ When this occurred, statistical models were constructed to include the exposure variable, the confound, and their interaction. Data analyses were performed from June 29, 2022, to June 23, 2023, using SPSS, version 29 (IBM Corp).

## Results

### Patient and Clinical Characteristics

The study population comprised 394 patients (mean [SD] age, 62.5 [11.5] years; 277 [70.3%%] males and 117 [29.7%] females) with head and neck cancer. Additional demographic and clinical characteristics are presented in Table 1. Race and ethnicity were not considered in the analysis.

**Table 1.** Clinical and Demographic Characteristics of Study Participants

Characteristic	No. (%)
Total participants, No.	394 (100)
Age at diagnosis, y	
Median (range)	61 (23-93)
Mean (SD)	62.5 (11.5)
Smoking, pack years	139 (35)
Median (range)	40.0 (0.5-180.0)
Mean (SD)	42.9 (28.8)
Female	117 (29.7)
Male	277 (70.3)
Site of disease	
Oral	143 (36.3)
Oropharyngeal	116 (29.4)
HPV^+^	73 (62.9)
HPV^−^	43 (37.1)
Laryngeal	84 (21.3)
Other site	51 (12.9)
T classification^a^	
T1-T2	101 (25.6)
T3-T4	290 (73.6)
Nodal metastases	
Negative	198 (49.9)
Positive	199 (50.1)
Treatment received	
Surgery only	55 (14.0)
Surgery plus RT	92 (23.4)
Surgery plus RT plus chemotherapy	47 (11.9)
RT and/or chemotherapy	200 (50.8)
PHQ−9 depression score	391 (99.0)
Median (range)	5.0 (0-27.0)
Mean (SD)	6.5 (6.2)
Systemic Inflammation Index	292 (74)
Median (range)	840.1 (48.8-13 525.9)
Mean (SD)	1264.9 (1394.8)
Incomplete tumor response	157 (39.8)
Deaths at 2 year follow-up	136 (34.5)
Two-year overall survival, mo	
Median (range)	24.0 (0-24.0)
Mean (SD)	17.7 (8.5)

Abbreviations: HPV, human papillomavirus; PHQ−9, Patient Health Questionnaire−9 item; RT, radiation therapy; T classification, tumor stage classification per the *American Joint Commission on Cancer Staging System, eighth edition*.

^a^ Tumor stage data were missing for 3 patients with complex clinical presentations and treatment courses; these tumors were never staged.

Among the 394 participants, 285 (72.3%) scored below the clinical cutoff for depression on the PHQ−9 (ie, score <9), and 157 patients (39.8%) exhibited an incomplete tumor response. We followed every patient for 2 years after study entry or until death. None were lost to follow-up—when a participant’s date of death was not available in our clinical records, data were collected from outside medical records and/or obituaries.

### Primary Analyses

Depressive symptoms at study entry were significantly associated with subsequent 2-year overall survival, and significantly associated with both SII and tumor response (Table 2). Both SII and tumor response were significantly associated with subsequent 2-year overall survival (SII: HR, 1.36; 95% CI, 1.08-1.71; tumor response: HR, 9.31; 95% CI, 6.24-13.91). SII was not associated with subsequent tumor response (odds ratio [OR], 1.32; 95% CI, 0.99-1.76).

**Table 2.** Tests of Total Associations of Depression and Indirect Association of Potential Mediators (Inflammation, Tumor Response to Treatment) With Overall Survival

Variable	Outcome	Hazard ratio (95% CI)
**Total associations**		
Depression	Overall survival	1.04 (1.02-1.07)
**Depression × mediator associations**		
Depression, partial *r* (95% CI)	Systemic inflammation	0.168 (0.007-0.038)
Depression, odds ratio (95% CI)	Tumor response	1.05 (1.01-1.08)
**Mediation associations**		
Mediation model 1		
Depression	Overall survival	1.08 (0.83-1.41)
Systemic inflammation		1.28 (1.00-1.64)
Depression** × **inflammation		0.99 (0.96-1.03)
Mediation model 2		
Depression	Overall survival	1.03 (1.00-1.06)
Tumor response		9.44 (6.23-14.32)
Depression** × **response		0.96 (0.91-1.02)

All variables were centered before analyses. Tests of mediation on the relation between depression and survival were performed with the MacArthur approach^30^ in which variables were centered and interaction terms were included. To show that Y mediates X on Z, it must be shown that X, Y, and Z happen in that order, that X and Y are correlated, and that Y explains all (complete) or part (partial) of the association between X and Z. In a linear model, this means that the main association of Y, the interaction between X and Y, or both are statistically significant.

**Figure 2.**
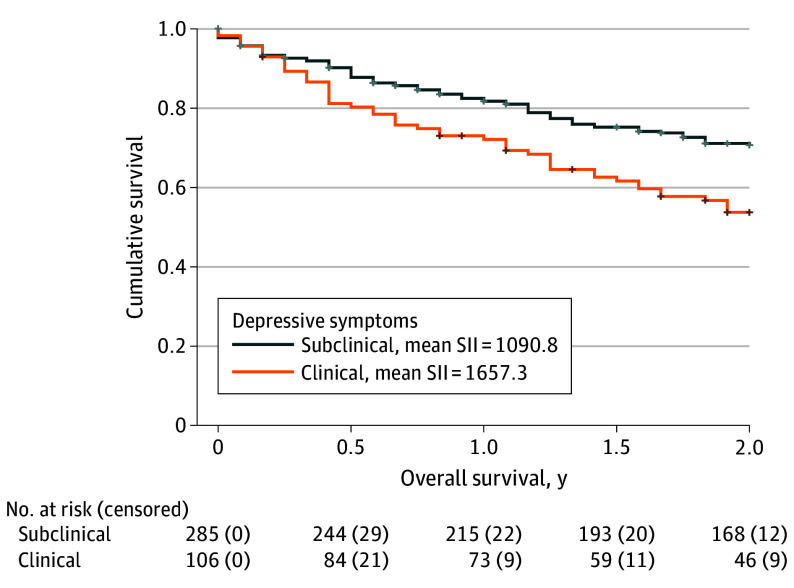
The Association of Depression on Overall Survival Mediated by Systemic Inflammation and Tumor Response Patients with clinically significant depressive symptom scores had higher systemic inflammation indices (SII), higher rates of cancer treatment failure and experienced earlier all-cause mortality. Kaplan-Meier curves depict all-cause mortality for patients with clinical vs subclinical depressive symptom scores. Analyses suggest that early mortality may have been mediated by 2 distinct pathways: through full mediation effects by SII (n = 292) and partial mediation effects by tumor response (n = 394).

When entered into the mediation model, the direct associations of depressive symptoms with overall survival lost significance, while the direct association of SII remained significant, indicating the association between depressive symptoms and 2-year overall survival was fully mediated by SII (Figure 2). In the second mediation model, the direct associations of both depressive symptoms and tumor response were significant, indicating that tumor response partially mediated the depression-survival association (Table 2).

#### Secondary Analyses

Of the possible confounding variables measured, the only variable associated both with depressive symptoms and overall survival was age at diagnosis. We ran a Cox proportional hazards model entering depressive symptoms, age, and their interaction: direct associations of depressive symptoms remained significantly associated with overall survival; the direct association of age and the association of the interaction term of age and depression were not significant. The only potential confounder associated with both SII and overall survival was T classification. When entered into the Cox model, direct associations of T classification were found to be significant, and the direct association of SII was lost (Table 3). Lastly, potentially confounding variables associated with both tumor response and overall survival included human papillomavirus status, cancer site, and T classification. In each of the separate models assessing the effects of these potential confounders, direct associations of tumor response remained significantly associated with overall survival (Table 3).

**Table 3.** Results After Adjustment for Confounding Variables Associated With Both the Exposure and Outcome Variables

Exposure	Outcome	HR (95% CI)
Systemic inflammation	Overall survival	0.57 (0.25-1.27)
T classification		0.01 (0-1.72)
Inflammation** × **T classification		2.45 (1.06-5.68)

Abbreviation: T classification, tumor stage classification per the *American Joint Commission on Cancer Staging System, eighth edition*.

## Discussion

Head and neck cancer diagnosis and treatment exact stress on the patient that increases vulnerability to depression.^32,33^ Patients with cancer who experience depressive symptoms are known to die early—an association that has been observed across multiple cancer types,^34,35,36^ notably including head and neck cancer.^3,24,37,38,39^ We previously reported on a cohort of 134 patients with head and neck cancer among whom depressive symptoms were associated with early mortality.^3^ We explored factors of cancer treatment as mediators of the depression-survival link, finding that tumor response partly explained the prognostic value of depression.^3^ Those findings pointed to the need for research to explore pathways 1 and 2 in addition to pathway 3 (Figure 1). Because of clearly established connections between systemic inflammation and depression,^10,11^ inflammation is a strong candidate mechanism within pathway 1. In this current new and larger cohort, we included SII to test pathway 1 in a prospective observational design during 2 years after diagnosis and treatment planning. Among this group of 394 patients with head and neck cancer, SII emerged as a statistical and plausible biological pathway (ie, mediator) of depression associations with cancer survival. Supporting the findings from clinical cancer data,^40,41^ younger patients reported more depressive symptoms than older patients, and they did not survive as long. However, after adjustment for age at diagnosis, depressive symptoms remained significantly associated with overall survival. Furthermore, age was not a confounding variable for inflammation given that age was not statistically associated with SII.^31^

In this larger cohort, we again observed that depressive symptoms were significantly associated with tumor response to treatment. As in our previous study,^3^ tumor response partially mediated the prognostic effect of depressive symptoms on survival. Although depression can impair a patient’s ability to complete taxing cancer treatments, this finding should be considered in the context of previous research showing that the depression-tumor response effect is not explained simply by treatment adherence.^3,42,43,44,45,46^ Together, these results indicate a need for future research that will illuminate potential biological mechanisms of the effects of psychosocial factors on tumor response to treatment, per se. Future research should evaluate the biological processes that may underlie associations of depression with treatment response and survival, such as sympathetic activation, hypothalamic-pituitary-adrenal (HPA) axis, rhythmicity, and measures of circadian disruption.^24,47,48^

SII was not significantly associated with tumor response. More precise biological measures of inflammation are needed in future studies. Research should consider other systemic biomarkers (eg, C-reactive protein, TNF-α, IL-1β, IL-6, and HPA axis dysregulation) as well as factors of the tumor microenvironment that may explain poor tumor response to treatment in patients with depressive symptoms.^48,49^ Given the strong associations of depression with neuroendocrine and immune changes, it is also reasonable to posit that depression may alter tumor response to treatment and cancer progression via peripheral cellular and molecular psychoneuroimmune changes. It is possible that both depression and cancer-related inflammation are secondary to β-adrenergic activation and HPA axis dysregulation.^50,51^ The stress hormone norepinephrine is known to induce resistance of oral cancer cells to cisplatin.^52^ Activated β-adrenergic pathways increase tumor cell proliferation, adhesion, invasion, and angiogenesis; and are associated with early mortality in patients with larynx and pharynx squamous cell carcinomas.^53^ Clinical trial results suggest serotonin should also be measured in this context, as serotonin-targeting drugs are less effective when used among patients with cancer who are depressed and have inflammation.^54,55^ New evidence also implicates serotonin-modulating genetic variants in oropharyngeal cancer aggressiveness and prognosis.^56^ Kynurenine pathways, thought to associate depression with inflammation through the brain glutamate receptors, may also be relevant.^57^ Relationships between depression and inflammation may also be directional, and should be tested; eg, chronic systemic inflammation secondary to tumor progression may cause immune signaling to the brain that leads to sickness behavior and depression—possibly explaining the elevated levels of depression among patients with cancer.^58^ New research should also extend to other tumor types given that inflammation is a risk factor for progression^59,60,61,62^ and survival across prostate, ovarian, breast, and head and neck cancers.^18,63,64,65,66,67^

Our tests for potentially confounding variables revealed that the direct association of SII with survival was not robust when T classification was entered into the model. It is possible that worsening inflammation concomitant with tumor progression may introduce covariance sufficient to cloud the relationships between inflammation, T classification, and survival. Larger tumors are capable of elevating host systemic inflammatory profiles.^18,20^ In turn, host inflammation may promote tumor growth.^68^ It is also difficult to disentangle the effects of host depression compared with the effects of the tumor on systemic inflammation. Additional prospective longitudinal research is needed to better differentiate tumor from host inflammatory profiles, any potential reciprocal effects, and how treatments aimed at mitigating inflammation may be associated with head and neck tumor growth and patient survival.

Somatic symptoms of depression often overlap with those of cancer, making it difficult to discern whether diagnostic criteria for depression are met. However, the current data clearly show a negative association with cancer outcomes, even for patients with depressive symptoms scoring less than the diagnostic threshold.^3^ To better understand this, we separated PHQ−9 scores into cognitive or affective and somatic symptom clusters. Post hoc Cox regressions revealed that both cognitive or affective and somatic depression subscales were significantly associated with overall survival. Depression associations with survival extended beyond merely physical symptoms (eg, fatigue) that may be spurred by the tumor. They included cognitive and/or affective components of depression with important translational implications for patients with head and neck cancer. Therefore, affective symptom management should be targeted irrespective of diagnostic qualification.

The clinical implications of this research are of paramount importance. Prompt diagnosis of depression should be prioritized in clinical settings. Depression screening should occur at multiple points along the treatment trajectory because newly developing symptoms can significantly and detrimentally affect cancer outcomes.^3,39,69^ Pharmacologic interventions have shown some promise in the treatment of depression in patients with head and neck cancer. For example, prophylactic escitalopram treatment has been shown to reduce the risk of developing depression and helped patients maintain quality of life.^70,71^ However, despite improvements that may be realized in affective and somatic symptoms, pharmacologic treatment does not affect the management of negative thoughts nor does it improve patients’ coping strategies for the challenges of cancer and its taxing treatments. Adverse effects and interactions with other therapeutics may limit patients’ tolerance of pharmacologic intervention, and some patients may simply be reluctant to add to their medication regimen. On the other hand, behavioral interventions can teach patients to become more adept at identifying depressive symptoms and responding with adaptive coping mechanisms, potentially allowing them to gain a sense of control over their experience.^72^

An approach with demonstrated effectiveness for the treatment of depression is that of collaborative care interventions in which an oncologist-led team offers assessment and intervention as needed.^73^ These teams use validated measures to track a patient’s progress along the cancer treatment trajectory. Treatments such as cognitive-behavioral therapy are then modified based on objective responses, allowing more patients to advance through appropriate treatment faster and more systematically than they otherwise would through usual care. This stepped-care approach allows professionals to tailor interventions according to the patient’s level of depressive symptoms. This approach has shown promise for decreasing health care resource utilization and cost,^74^ which is especially important for patients with low income and/or of minority groups who may have limited access to care. Given that the availability of collaborative care models remains limited, further study is needed to determine how best to implement care models on a widespread, feasible, and sustainable basis, especially among patients with low income and populations in rural areas.^75,76^

### Limitations

A limitation of this research was the absence of repeated measures to identify the trajectory of depression over time, which would better pinpoint the spikes in symptoms and the most opportune times for intervention. More data will also be required to determine whether benefits of interventions for depression are sustained over time. In addition, although the SII is readily available and easy to obtain, varying prognostic thresholds have been reported in the literature^19,22^ and an optimal cutoff for potential clinical intervention has not been established. There was also some variability in treatment status at the time of study initiation: some patients had already undergone a surgical procedure before collection of depression and SII data. Moreover, SII data were not available for all patients given that we relied on clinical standards of care to determine whether these data would be collected. The use of tumor response may have some limitations because it can often be difficult to distinguish recurrences from new primary disease at the time of evaluation for tumor response. New primary tumors may be fairly common in head and neck cancer, although review of medical records showed that only 1 patient in our sample evidenced a distant new primary tumor at the 6-month follow-up. In addition, treatment-related toxic effects were not assessed and could be associated with subsequent tumor response.

## Conclusions

This longitudinal study points to systemic inflammation as a plausible biological pathway by which depression affects head and neck cancer survival (pathway 1, Figure 1). This study replicates prior findings demonstrating significant associations between depression and overall survival among patients with head and neck cancer. These findings provide important and novel data suggesting that systemic inflammation may mediate the depression-survival relationship in head and neck cancer. Our data underscore the notion that depressive symptoms may have powerful effects on par with the clinical features typically used to understand patient prognosis. Screening and early efficacious treatment for even mild depressive symptoms have the potential to reduce the human cost of head and neck cancer.

## Notes

Supplement. Data Sharing Statement

## References

[1] Massie MJ. Prevalence of depression in patients with cancer. J Natl Cancer Inst Monogr. 2004;(32):57-71. doi:10.1093/jncimonographs/lgh014 15263042

[2] Osazuwa-Peters N, Boakye EA, Mohammed KA, . Prevalence and sociodemographic predictors of depression in patients with head and neck cancer: results from a national study. J Clin Oncol. 2016;34(15)(suppl):6064-6064. doi:10.1200/JCO.2016.34.15_suppl.6064

[3] Zimmaro LA, Sephton SE, Siwik CJ, . Depressive symptoms predict head and neck cancer survival: examining plausible behavioral and biological pathways. Cancer. 2018;124(5):1053-1060. doi:10.1002/cncr.31109 29355901 PMC5821545

[4] Miller AH. Beyond depression: the expanding role of inflammation in psychiatric disorders. World Psychiatry. 2020;19(1):108-109. doi:10.1002/wps.20723 31922681 PMC6953590

[5] Troubat R, Barone P, Leman S, . Neuroinflammation and depression: a review. Eur J Neurosci. 2021;53(1):151-171. doi:10.1111/ejn.14720 32150310

[6] Irwin M. Psychoneuroimmunology of depression: clinical implications. Brain Behav Immun. 2002;16(1):1-16. doi:10.1006/brbi.2001.0654 11846437

[7] Leonard BE, Myint A. The psychoneuroimmunology of depression. Hum Psychopharmacol. 2009;24(3):165-175. doi:10.1002/hup.1011 19212943

[8] Rengasamy M, Marsland A, Spada M, Hsiung K, Kovats T, Price RB. A chicken and egg scenario in psychoneuroimmunology: bidirectional mechanisms linking cytokines and depression. J Affect Disord Rep. 2021;6:6. doi:10.1016/j.jadr.2021.100177 35992016 PMC9387766

[9] Sforzini L, Nettis MA, Mondelli V, Pariante CM. Inflammation in cancer and depression: a starring role for the kynurenine pathway. Psychopharmacology (Berl). 2019;236(10):2997-3011. doi:10.1007/s00213-019-05200-8 30806743 PMC6820591

[10] Raedler TJ. Inflammatory mechanisms in major depressive disorder. Curr Opin Psychiatry. 2011;24(6):519-525. doi:10.1097/YCO.0b013e32834b9db6 21897249

[11] Raison CL, Capuron L, Miller AH. Cytokines sing the blues: inflammation and the pathogenesis of depression. Trends Immunol. 2006;27(1):24-31. doi:10.1016/j.it.2005.11.006 16316783 PMC3392963

[12] Strawbridge R, Arnone D, Danese A, Papadopoulos A, Herane Vives A, Cleare AJ. Inflammation and clinical response to treatment in depression: a meta-analysis. Eur Neuropsychopharmacol. 2015;25(10):1532-1543. doi:10.1016/j.euroneuro.2015.06.007 26169573

[13] Antoni MH, Dhabhar FS. The impact of psychosocial stress and stress management on immune responses in patients with cancer. Cancer. 2019;125(9):1417-1431. doi:10.1002/cncr.31943 30768779 PMC6467795

[14] Qian S, Golubnitschaja O, Zhan X. Chronic inflammation: key player and biomarker-set to predict and prevent cancer development and progression based on individualized patient profiles. EPMA J. 2019;10(4):365-381. doi:10.1007/s13167-019-00194-x 31832112 PMC6882964

[15] Cui S, Li J, Liu Y, . Correlation of systemic immune-inflammation index and moderate/major depression in patients with depressive disorders: a large sample cross-sectional study. Front Psychiatry. 2023;14:1159889. doi:10.3389/fpsyt.2023.1159889 37275977 PMC10232846

[16] Zhu X, Li R, Zhu Y, . Neutrophil/lymphocyte, platelet/lymphocyte, monocyte/lymphocyte ratios and systemic immune-inflammation index in patients with depression. Bratisl Lek Listy. 2023;124(6):471-474. doi:10.4149/BLL_2023_072 36876383

[17] Feng JF, Chen S, Yang X. Systemic immune-inflammation index (SII) is a useful prognostic indicator for patients with squamous cell carcinoma of the esophagus. Medicine (Baltimore). 2017;96(4):e5886. doi:10.1097/MD.0000000000005886 28121932 PMC5287956

[18] Wang YT, Kuo LT, Weng HH, . Systemic Immune-Inflammation Index as a predictor for head and neck cancer prognosis: a meta-analysis. Front Oncol. 2022;12:899518. doi:10.3389/fonc.2022.899518 35814369 PMC9263088

[19] Atasever Akkas E, Yucel B. Prognostic value of systemic immune inflammation index in patients with laryngeal cancer. Eur Arch Otorhinolaryngol. 2021;278(6):1945-1955. doi:10.1007/s00405-021-06798-2 33837464

[20] Diao P, Wu Y, Li J, . Preoperative systemic immune-inflammation index predicts prognosis of patients with oral squamous cell carcinoma after curative resection. J Transl Med. 2018;16(1):365. doi:10.1186/s12967-018-1742-x 30563540 PMC6299596

[21] Hung SP, Chen PR, Ho TY, . Prognostic significance of the preoperative systemic immune-inflammation index in patients with oral cavity squamous cell carcinoma treated with curative surgery and adjuvant therapy. Cancer Med. 2021;10(2):649-658. doi:10.1002/cam4.3650 33325655 PMC7877364

[22] Li Z, Qu Y, Yang Y, . Prognostic value of the neutrophil-to-lymphocyte ratio, platelet-to-lymphocyte ratio and systemic immune-inflammation index in patients with laryngeal squamous cell carcinoma. Clin Otolaryngol. 2021;46(2):395-405. doi:10.1111/coa.13689 33321001

[23] Hu B, Yang XR, Xu Y, . Systemic immune-inflammation index predicts prognosis of patients after curative resection for hepatocellular carcinoma. Clin Cancer Res. 2014;20(23):6212-6222. doi:10.1158/1078-0432.CCR-14-0442 25271081

[24] Cash E, Duck CR, Brinkman C, . Depressive symptoms and actigraphy-measured circadian disruption predict head and neck cancer survival. Psychooncology. 2018;27(10):2500-2507. doi:10.1002/pon.4862 30117225

[25] Kroenke K, Spitzer RL, Williams JB. The PHQ-9: validity of a brief depression severity measure. J Gen Intern Med. 2001;16(9):606-613. doi:10.1046/j.1525-1497.2001.016009606.x 11556941 PMC1495268

[26] Britton B, Clover K, Bateman L, . Baseline depression predicts malnutrition in head and neck cancer patients undergoing radiotherapy. Support Care Cancer. 2012;20(2):335-342. doi:10.1007/s00520-011-1087-y 21234608

[27] Shinn EH, Valentine A, Jethanandani A, . Depression and oropharynx cancer outcome. Psychosom Med. 2016;78(1):38-48. doi:10.1097/PSY.0000000000000256 26632757 PMC4696911

[28] Eisenhauer EA, Therasse P, Bogaerts J, . New response evaluation criteria in solid tumours: revised RECIST guideline (version 1.1). Eur J Cancer. 2009;45(2):228-247. doi:10.1016/j.ejca.2008.10.026 19097774

[29] Kraemer HC, Blasey CM. Centering in regression analyses: a strategy to prevent errors in statistical inference. Int J Methods Psychiatr Res. 2004;13(3):141-151. doi:10.1002/mpr.170 15297898 PMC6878533

[30] Kraemer HC, Kiernan M, Essex M, Kupfer DJ. How and why criteria defining moderators and mediators differ between the Baron & Kenny and MacArthur approaches. Health Psychol. 2008;27(2S):S101-S108. doi:10.1037/0278-6133.27.2(Suppl.).S101 18377151 PMC3376898

[31] Kraemer HC, Stice E, Kazdin A, Offord D, Kupfer D. How do risk factors work together? Mediators, moderators, and independent, overlapping, and proxy risk factors. Am J Psychiatry. 2001;158(6):848-856. doi:10.1176/appi.ajp.158.6.848 11384888

[32] Linden W, Vodermaier A, Mackenzie R, Greig D. Anxiety and depression after cancer diagnosis: prevalence rates by cancer type, gender, and age. J Affect Disord. 2012;141(2-3):343-351. doi:10.1016/j.jad.2012.03.025 22727334

[33] Rodin G, Lo C, Mikulincer M, Donner A, Gagliese L, Zimmermann C. Pathways to distress: the multiple determinants of depression, hopelessness, and the desire for hastened death in metastatic cancer patients. Soc Sci Med. 2009;68(3):562-569. doi:10.1016/j.socscimed.2008.10.037 19059687

[34] Pinquart M, Duberstein PR. Depression and cancer mortality: a meta-analysis. Psychol Med. 2010;40(11):1797-1810. doi:10.1017/S0033291709992285 20085667 PMC2935927

[35] Spiegel D, Giese-Davis J. Depression and cancer: mechanisms and disease progression. Biol Psychiatry. 2003;54(3):269-282. doi:10.1016/S0006-3223(03)00566-3 12893103

[36] Walker J, Mulick A, Magill N, . Major depression and survival in people with cancer. Psychosom Med. 2021;83(5):410-416. doi:10.1097/PSY.0000000000000942 33938501 PMC7614901

[37] Barber B, Dergousoff J, Slater L, . Depression and survival in patients with head and neck cancer: a systematic review. JAMA Otolaryngol Head Neck Surg. 2016;142(3):284-288. doi:10.1001/jamaoto.2015.3171 26796781

[38] Rieke K, Schmid KK, Lydiatt W, Houfek J, Boilesen E, Watanabe-Galloway S. Depression and survival in head and neck cancer patients. Oral Oncol. 2017;65:76-82. doi:10.1016/j.oraloncology.2016.12.014 28109472 PMC8201663

[39] Van der Elst S, Bardash Y, Wotman M, Kraus D, Tham T. The prognostic impact of depression or depressive symptoms on patients with head and neck cancer: a systematic review and meta-analysis. Head Neck. 2021;43(11):3608-3617. doi:10.1002/hed.26868 34525238

[40] Lang MJ, David V, Giese-Davis J. The age conundrum: a scoping review of younger age or adolescent and young adult as a risk factor for clinical distress, depression, or anxiety in cancer. J Adolesc Young Adult Oncol. 2015;4(4):157-173. doi:10.1089/jayao.2015.0005 26697266 PMC4684657

[41] Yi JC, Syrjala KL. Anxiety and depression in cancer survivors. Med Clin North Am. 2017;101(6):1099-1113. doi:10.1016/j.mcna.2017.06.005 28992857 PMC5915316

[42] DiMatteo MR, Haskard-Zolnierek KB. Impact of depression on treatment adherence and survival from cancer. In: Kissane DW, Maj M, Sartorius N, eds. Depression and cancer. Wiley-Blackwell; 2011:101-124.

[43] Arrieta O, Angulo LP, Núñez-Valencia C, . Association of depression and anxiety on quality of life, treatment adherence, and prognosis in patients with advanced non-small cell lung cancer. Ann Surg Oncol. 2013;20(6):1941-1948. doi:10.1245/s10434-012-2793-5 23263699

[44] Kissane D. Beyond the psychotherapy and survival debate: the challenge of social disparity, depression and treatment adherence in psychosocial cancer care. Psychooncology. 2009;18(1):1-5. doi:10.1002/pon.1493 19097139

[45] Mausbach BT, Schwab RB, Irwin SA. Depression as a predictor of adherence to adjuvant endocrine therapy (AET) in women with breast cancer: a systematic review and meta-analysis. Breast Cancer Res Treat. 2015;152(2):239-246. doi:10.1007/s10549-015-3471-7 26077640 PMC4861253

[46] Perlow HK, Ramey SJ, Cassidy V, . Disparities in adherence to head and neck cancer follow-up guidelines. Laryngoscope. 2019;129(10):2303-2308. doi:10.1002/lary.27676 30582620 PMC7757086

[47] Cash E, Sephton S, Woolley C, . The role of the circadian clock in cancer hallmark acquisition and immune-based cancer therapeutics. J Exp Clin Cancer Res. 2021;40(1):119. doi:10.1186/s13046-021-01919-5 33794967 PMC8017624

[48] Chang A, Sloan EK, Antoni MH, Knight JM, Telles R, Lutgendorf SK. Biobehavioral pathways and cancer progression: insights for improving well-being and cancer outcomes. Integr Cancer Ther. 2022;21:15347354221096081. doi:10.1177/15347354221096081 35579197 PMC9118395

[49] Miller AH, Raison CL. The role of inflammation in depression: from evolutionary imperative to modern treatment target. Nat Rev Immunol. 2016;16(1):22-34. doi:10.1038/nri.2015.5 26711676 PMC5542678

[50] Ahmad MH, Rizvi MA, Fatima M, Mondal AC. Pathophysiological implications of neuroinflammation mediated HPA axis dysregulation in the prognosis of cancer and depression. Mol Cell Endocrinol. 2021;520:111093. doi:10.1016/j.mce.2020.111093 33253761

[51] Furman D, Campisi J, Verdin E, . Chronic inflammation in the etiology of disease across the life span. Nat Med. 2019;25(12):1822-1832. doi:10.1038/s41591-019-0675-0 31806905 PMC7147972

[52] Tjioe KC, Cardoso DM, Oliveira SHP, Bernabé DG. Stress hormone norepinephrine incites resistance of oral cancer cells to chemotherapy. Endocr Relat Cancer. 2022;29(4):201-212. doi:10.1530/ERC-20-0460 35099408

[53] Lopes-Santos G, Bernabé DG, Miyahara GI, Tjioe KC. Beta-adrenergic pathway activation enhances aggressiveness and inhibits stemness in head and neck cancer. Transl Oncol. 2021;14(8):101117. doi:10.1016/j.tranon.2021.101117 33993095 PMC8236611

[54] Jha MK, Minhajuddin A, Gadad BS, . Can C-reactive protein inform antidepressant medication selection in depressed outpatients? findings from the CO-MED trial. Psychoneuroendocrinology. 2017;78:105-113. doi:10.1016/j.psyneuen.2017.01.023 28187400 PMC6080717

[55] Uher R, Tansey KE, Dew T, . An inflammatory biomarker as a differential predictor of outcome of depression treatment with escitalopram and nortriptyline. Am J Psychiatry. 2014;171(12):1278-1286. doi:10.1176/appi.ajp.2014.14010094 25017001

[56] Queiroz GSR, Carron J, Macedo LT, Lima CSP, Lourenço GJ. Influence of variants in serotonin modulating genes on the risk, aggressiveness, and prognosis of oropharynx cancer. Head Neck. 2023;45(7):1790-1800. doi:10.1002/hed.27394 37158249

[57] Haroon E, Welle JR, Woolwine BJ, . Associations among peripheral and central kynurenine pathway metabolites and inflammation in depression. Neuropsychopharmacology. 2020;45(6):998-1007. doi:10.1038/s41386-020-0607-1 31940661 PMC7162907

[58] Dantzer R, O’Connor JC, Freund GG, Johnson RW, Kelley KW. From inflammation to sickness and depression: when the immune system subjugates the brain. Nat Rev Neurosci. 2008;9(1):46-56. doi:10.1038/nrn2297 18073775 PMC2919277

[59] Archer M, Dogra N, Kyprianou N. Inflammation as a driver of prostate cancer metastasis and therapeutic resistance. Cancers (Basel). 2020;12(10):2984. doi:10.3390/cancers12102984 33076397 PMC7602551

[60] Khusnurrokhman G, Wati FF. Tumor-promoting inflammation in lung cancer: a literature review. Ann Med Surg (Lond). 2022;79:104022. doi:10.1016/j.amsu.2022.104022 35860063 PMC9289429

[61] Kraus S, Arber N. Inflammation and colorectal cancer. Curr Opin Pharmacol. 2009;9(4):405-410. doi:10.1016/j.coph.2009.06.006 19589728

[62] Neagu M, Constantin C, Caruntu C, Dumitru C, Surcel M, Zurac S. Inflammation: a key process in skin tumorigenesis. Oncol Lett. 2019;17(5):4068-4084. 30944600 10.3892/ol.2018.9735 PMC6444305

[63] Atasever Akkas E, Erdis E, Yucel B. Prognostic value of the systemic immune-inflammation index, systemic inflammation response index, and prognostic nutritional index in head and neck cancer. Eur Arch Otorhinolaryngol. 2023;280(8):3821-3830. doi:10.1007/s00405-023-07954-6 37029321

[64] Ji Y, Wang H. Prognostic prediction of systemic immune-inflammation index for patients with gynecological and breast cancers: a meta-analysis. World J Surg Oncol. 2020;18(1):197. doi:10.1186/s12957-020-01974-w 32767977 PMC7414550

[65] Meng L, Yang Y, Hu X, Zhang R, Li X. Prognostic value of the pretreatment systemic immune-inflammation index in patients with prostate cancer: a systematic review and meta-analysis. J Transl Med. 2023;21(1):79. doi:10.1186/s12967-023-03924-y 36739407 PMC9898902

[66] Qiu Y, Zhang Z, Chen Y. Prognostic value of pretreatment Systemic Immune-Inflammation Index in gastric cancer: a meta-analysis. Front Oncol. 2021;11:537140. doi:10.3389/fonc.2021.537140 33777726 PMC7990885

[67] Atasever Akkas E, Erdis E, Yucel B. Prognostic value of the systemic immune-inflammation index, systemic inflammation response index, and prognostic nutritional index in head and neck cancer. Eur Arch Otorhinolaryngol. 2023;280(8):3821-3830. doi:10.1007/s00405-023-07954-6 37029321

[68] Wilkie KP, Aktar F. Mathematically modelling inflammation as a promoter of tumour growth. Math Med Biol. 2020;37(4):491-514. doi:10.1093/imammb/dqaa005 32430508

[69] Korsten LHA, Jansen F, de Haan BJF, . Factors associated with depression over time in head and neck cancer patients: a systematic review. Psychooncology. 2019;28(6):1159-1183. doi:10.1002/pon.5058 30865357 PMC6593868

[70] Lydiatt WM. Improved quality of life and function after oropharyngeal cancer treatment. JAMA Otolaryngol Head Neck Surg. 2013;139(11):1108-1109. doi:10.1001/jamaoto.2013.2774 23575518

[71] Panwar A, McGill T, Lydiatt D, . De-novo depression, prophylactic antidepressant, and survival in patients with head and neck cancer. Laryngoscope. 2023;133(4):856-862. doi:10.1002/lary.30249 35730719 PMC10321851

[72] Spiegel D. Mind matters in cancer survival. Psychooncology. 2012;21(6):588-593. doi:10.1002/pon.3067 22438289 PMC3370072

[73] Li M, Kennedy EB, Byrne N, . Systematic review and meta-analysis of collaborative care interventions for depression in patients with cancer. Psychooncology. 2017;26(5):573-587. doi:10.1002/pon.4286 27643388

[74] Duarte A, Walker J, Walker S, . Cost-effectiveness of integrated collaborative care for comorbid major depression in patients with cancer. J Psychosom Res. 2015;79(6):465-470. doi:10.1016/j.jpsychores.2015.10.012 26652589 PMC4678258

[75] Noel CW, Sutradhar R, Chan WC, . Gaps in depression symptom management for patients with head and neck cancer. Laryngoscope. 2023;133(10):2638-2646. doi:10.1002/lary.30595 36748910

[76] Richardson AE, Broadbent E, Morton RP. A systematic review of psychological interventions for patients with head and neck cancer. Support Care Cancer. 2019;27(6):2007-2021. doi:10.1007/s00520-019-04768-3 30937599

