# Supplementary material for: Depressive Symptoms, Systemic Inflammation, and Survival Among Patients With Head and Neck Cancer
Source: JAMA Otolaryngol Head Neck Surg. 2024 Mar 28;150(5):405–13. doi: 10.1001/jamaoto.2024.0231 (PMC10979366; doi:10.1001/jamaoto.2024.0231)
Supplement: Supplement. — Data Sharing Statement [file jamaotolaryngolheadnecksurg-e240231-s001.pdf]

Data Sharing Statement

Cash. Depressive Symptoms, Systemic Inflammation, and Survival Among Patients With Head and Neck Cancer. *JAMA Otolaryngol Head Neck Surg*. Published March 28, 2024. doi:10.1001/jamaoto.2024.0231

Data
Data available: Yes
Data types: Deidentified participant data
How to access data: The data underlying this article are available in OpenICPSR.org, at https://dx.doi.org/10.3886/E193207V1
When available: beginning date: 08-01-2025

Supporting Documents
Document types: Statistical/analytic code
How to access documents: The supporting documents underlying this article are available in OpenICPSR.org, at https://dx.doi.org/10.3886/E193207V1
When available: beginning date: 08-01-2025

Additional Information
Who can access the data: The data underlying this article are available in OpenICPSR.org, at https://dx.doi.org/10.3886/E193207V1
Types of analyses: None.
Mechanisms of data availability: Without investigator support.
